# Cross-linguistic effects of the speech-to-song illusion in speakers of Bangla and English

**DOI:** 10.1177/17470218241293627

**Published:** 2024-12-09

**Authors:** Rakhi Akter, Alexis Deighton MacIntyre

**Affiliations:** 1 Institute of Cognitive Neuroscience, University College London, London, UK; 2 MRC Cognition and Brain Sciences Unit, University of Cambridge, Cambridge, UK

**Keywords:** Auditory perception, cross-cultural perception, music perception, speech perception, Bangla language

## Abstract

The speech-to-song illusion is a phenomenon in which the continuous repetition of a spoken utterance induces the listeners to perceive it as more song-like. Thus far, this perceptual transformation has been observed in mostly European languages, such as English; however, it is unclear whether the illusion is experienced by speakers of Bangla (Bengali), an Indo-Aryan language. The current study, therefore, investigates the illusion in 28 Bangla and 31 English-speaking participants. The experiment consisted of a listening task in which participants were asked to rate their perception of repeating short speech stimuli on a scale from 1 to 5, where 1 = *sounds like speech* and 5 = *sounds like song*. The stimuli were composed of English and Bangla utterances produced by two bilingual speakers. To account for possible group differences in music engagement, participants self-reported musical experience and also performed a rhythm discrimination task as an objective measure of non-verbal auditory sequence processing. Stimulus ratings were analysed with cumulative link mixed modelling. Overall, English- and Bangla-speaking participants rated the stimuli similarly and, in both groups, better performance in the rhythm discrimination task significantly predicted more song-like ratings beyond self-reported musical experience. English speakers rated Bangla stimuli as significantly more song-like than English stimuli. Bangla speakers did not distinguish between English and Bangla stimuli—possibly reflecting their enhanced understanding of English, in comparison to the English participants’ comprehension of Bangla. An exploratory acoustic analysis revealed the role of harmonic ratio in the illusion for both language groups. These results demonstrate that the speech-to-song-illusion occurs for Bangla speakers to a similar extent as English speakers and that, across both groups, sensitivity to non-verbal auditory structure is positively correlated with susceptibility to this perceptual transformation.

## Introduction

The speech-to-song illusion is a perceptual transformation wherein hearing multiple repetitions of a spoken utterance result in it being perceived as more musical, or having a song-like quality (Deutsch et al., 2011). The transformation can take place without any manipulation of the speech audio signal, nor without any specific training to the listener (Vanden Bosch der Nederlanden et al., 2015); however, acoustic and phonological aspects of the utterance may increase the likelihood of inducing the illusion (Falk et al., 2014; Rathcke et al., 2021; Tierney et al., 2018). Classifying human vocally produced sounds as speech versus song has long been of interest to ethnomusicologists. As List (1963) notes:

certain cultures make a distinction between what is referred to as speech or talking and what is referred to as song or singing. Other cultures do not necessarily make this distinction [. . .] The nomenclature applied to [intermediate] forms will vary considerably from culture to culture, as will the social function of the form.

For researchers in brain and behavioural sciences, the illusion provides an opportunity to investigate perception across domains while holding the physical properties of the stimulus itself constant, providing insight into how internal processes shape our experience of the environment (der Nederlanden et al., 2015). Yet, the phenomenon is typically examined with participants recruited from North American or European university communities (e.g., Margulis et al., 2015; Rathcke et al., 2021; Vitevitch et al., 2023). It is, therefore, possible that previously described effects do not generalise to linguistic and/or cultural contexts outside of these populations. For example, one study reported that speakers of the tonal languages Thai and Mandarin experienced weaker illusory effects in comparison to speakers of German and Italian, which are non-tonal languages (Jaisin et al., 2016); however, this finding recently failed to replicate in a much larger sample with Mandarin and Cantonese speakers (Kachlicka et al., 2024). Culturally specific aspects of the stimuli may also affect experience of the illusion; for instance, Western listeners were more sensitive to acoustic manipulations that violated, rather than conformed to, European musical norms (der Nederlanden et al., 2015).

Without broadening the diversity of participants studied thus far, it is difficult to ascertain which aspects of the speech-to-song illusion are universal, and which are specific to particular populations. For instance, it is yet to be empirically established that the illusion occurs within a South Asian cultural and linguistic context. Although Margulis et al. (2015) employed Hindi stimuli, that language was unfamiliar to the English-speaking participants in their study. Bangla (Bengali) is another Indo-Aryan language that is insufficiently studied within psychology and neuroscience generally, despite being the seventh most widely spoken language globally (~ 273 million speakers; Central Intelligence Agency 2024). It is the official language of Bangladesh, and is also spoken in parts of India and many diaspora communities around the world. Unlike Thai or Mandarin, which were studied previously with regard to the illusion (Jaisin et al., 2016; Kachlicka et al., 2024), Bangla does not employ lexical tones, making it more similar to European languages like English, which is well studied in the context of the speech-to-song illusion. On the contrary, Bangla and English diverge in important ways: For instance, whereas English speech rhythm is characterised by alternating patterns of strong and weakly stressed syllables, marked by differences in intensity and duration (among other cues), the analogous prosodic unit in Bangla consists of alternating accented and unaccented changes in pitch (Khan, 2014). Bangla and English do share other suprasegmental properties in common, however, such that adult speakers emphasise similar intonational features when addressing infants in their own language (e.g., by increasing vocal range or exaggerating F0 contours; Yu et al., 2014). In sum, although English and Bangla are alike in some respects, it remains to be seen whether Bangla speakers perceive the illusion or respond to specific stimuli similarly to English speakers.

To understand the mechanisms underlying the speech-to-song illusion, it is critical to establish that the perceptual transformation is applicable to groups outside the populations typically centred by Western psychology and neuroscience (Blasi et al., 2022; Vitevitch et al., 2014). Towards this goal, the current report describes a perceptual experiment investigating the speech-to-song illusion in Bangla and English. Using an online paradigm, we recruited speakers of each language to perform a listening task in which they rated short, repeating phrases according to how speech-like or song-like they sounded. Both groups were presented with the same stimuli set, which consisted of Bangla and English speech excerpts produced by two Bangla–English bilingual speakers. In addition to completing the illusion listening task, the participants also answered a brief demographics questionnaire detailing their personal engagement with music, and performed a musical rhythm discrimination task as an objective, non-verbal measure of sensitivity to auditory sequential structure. This information was collected to gauge possible differences in musical experience that could affect group-level differences in the illusion listening task.

At the outset, it is important to note that we could not recruit Bangla-speaking participants who were exactly matched with the English-speaking participants, whether in terms of exposure to English, or monolingualism: English is widely spoken and understood in Bangladesh, a former British colony (Ministry of Foreign Affairs, The Government of Bangladesh, 2024), and most people in South Asia communicate using multiple languages in daily life (Bhatia & Ritchie, 2006). On the contrary, although English instruction is compulsory in Bengali primary and secondary schools, attainment varies both regionally and socioeconomically, and the national average ability is estimated as poor, though improving (Ara, 2020; Chowdhury & Kabir, 2014; Education First, 2024). The Bangla-speaking participants in the current study self-reported limited knowledge of English, but the data presented here and the interpretation thereof should be considered with this uncertainty and imbalance between the language groups in mind.

### Aims and hypothesis

In this experiment, our primary aim was to compare perception of the speech-to-song illusion in Bangla speakers to English speakers, with susceptibility to the speech-to-song illusion well established in the latter group. Given recent evidence showing that a large sample of Mandarin and Cantonese speakers responded similarly to English speakers for English speech stimuli (Kachlicka et al., 2024), we anticipated that Bangla-speaking listeners would report the perceptual transformation to a similar extent as English-speaking listeners. As a secondary research aim, we were also interested in whether the two language groups would rate stimuli from the other language as more song-like in comparison to their primary language. This follows from previous work indicating a stronger or more song-like effect of less familiar languages (Castro et al., 2018; Jaisin et al., 2016; Margulis et al., 2015; Rathcke et al., 2021). We predicted that English-speaking participants would rate Bangla language stimuli as more song-like, with the reverse pattern observed for Bangla-speaking participants—bearing in mind that the Bangla speakers in this study are likely to have more familiarity with English than vice versa. We discuss this complexity in more detail in the “Materials and methods” section.

Although the speech-to-song illusion was originally described in a musically trained sample (Deutsch et al., 2011), later experiments showed that formal training was not required to evoke the transformation (Vanden Bosch der Nederlanden et al., 2015). Nonetheless, individual differences in music perception and ability may modulate the tendency to perceive musicality in speech, or enhance the strength of the transformation (i.e., increase the contrast between speech-like and song-like; Tierney et al., 2021). Tierney et al. (2018) directly tested participants’ musical aptitude using a variety of batteries. They found that a musical timing task, the Beat Alignment Test (Iversen & Patel, 2008), best predicted illusion ratings in their sample; however, participants’ judgements of melody in a separate task were also positively correlated with more intense experience of the illusion (Tierney et al., 2021). Hence, as potential baseline differences in musical activities between the Bangla- and English-speaking participants could affect their responses in the illusion task, we collected self-reported measures of music engagement in everyday life. Existing inventories, such as the Goldsmiths Musical Sophistication Index (Müllensiefen et al., 2014), were not available as Bangla versions, so a novel questionnaire was developed by the authors to be culturally inclusive (e.g., not specific to the European conservatory tradition of musical training) and suitably brief for online data collection. In addition, we administered an auditory rhythm discrimination task that predicted sensitivity to speech prosody in a previous study (MacIntyre & Scott, 2022). The rhythm discrimination task asks listeners whether an additional presentation of a pattern of non-pitched drum sounds is the same or different from the previous presentation. Like the Beat Alignment Test, the rhythm discrimination task probes listeners’ aptitude in the temporal, rather than spectral (e.g., pitch), domain. Yet, distinguishing between the irregular patterning of drum sound onsets also requires the ability to process sequences in working memory. In this way, the task combines aspects of both rhythm and melody perception. As sensitivity within both these domains were found to predict speech-to-song illusion perception (Tierney et al., 2018), we hypothesised that higher scores on the rhythm discrimination task would positively correlate with more song-like ratings in the current study.

### Exploratory acoustic analysis

The current study was not designed to probe detailed acoustic or linguistic (e.g., syllabic timing) differences between Bangla and English that could interact with the speech-to-song-illusion. For example, the stimuli were produced by two speakers of South Asian English, an accent group that shares some phonological characteristics with other languages spoken in the Indian subcontinent (Wiltshire, 2020). Thus, although syllabic stress is realised in Bangla-accented English, the degree of differentiation between stressed and unstressed syllables is reduced when compared to English spoken in European regions (Saha & Das Mandal, 2016). With that said, we took the opportunity to perform an exploratory analysis of particular features of the stimuli that may be relevant for the illusion. These include the normalised Pairwise Variability Index (nPVI; Kachlicka et al., 2024; Nolan & Asu, 2009; Tierney et al., 2018), which describes the variability in timing of successive syllables, and sonority (Rathcke et al., 2021), which we estimated acoustically using the signal harmonic-to-noise ratio. We also quantified variability of vocal pitch, which may be pertinent for Bangla stimuli, given the phonological prominence of tonal sequences in that language (Khan, 2014).

## Materials and methods

### Participants

#### Sample size

Using a mixed effects model, Rathcke et al. (2021) found language-based differences, *z* = 4.78, *p* < 0.001 in a sample of *n* = 80 (40 English speakers and 40 French speakers); however, traditional effect sizes are not straightforward to calculate when the data are hierarchically structured, and software for Monte Carlo simulation-based power analyses are not yet widely available for ordinal-dependent variables (Green & MacLeod, 2016). A simulation-based analysis with a comparable design and outcome variable to our study, however, showed that two groups with 20 participants each providing six paired observations per participant are sufficient to detect *d* = 0.40 with 75–85% power (Hadavi et al., 2023). Extrapolating from this estimate, and given our design involves a two-way factorial interaction; our target was 60 participants (30 per language group) providing 12 ratings per stimulus language or 24 ratings in total.

#### Recruitment and demographics

Subjects were aged 18–40 years (English M = 31.58, SD = 4.99; Bangla M = 28.29, SD = 5.00) and reported no history of hearing or speech and language-related disorders, as well as never having lived abroad. English speakers were mostly based in the United Kingdom, with one participant from Australia and one from South Africa; Bangla speakers were based in Bangladesh and India.

Whereas the English-speaking participants were recruited online using Prolific (Palan & Schitter, 2018), Bangla speakers reporting limited knowledge of English were virtually absent from that platform. We, therefore, shifted to informal networks and word of mouth to recruit the Bangla-speaking sample. Before participating in the online experiment, individuals confirmed via email that they did not speak English and had received minimal education in English. As we recruited adult participants who had not undertaken any formalised assessments in English, it was infeasible to determine their level of fluency as it would be conventionally understood within regions like the United Kingdom; however, given the colonial legacy in Bangladesh and global prevalence of English today, we can expect at least passing familiarity with English for most Bangla speakers, including those in the current study.

To ensure high-quality data in the case of the English speakers, we screened for established participants with consistently high data acceptance rates on Prolific. As we recruited and interacted with the Bangla speakers directly, we were confident that they were motivated and attentive participants. According to their pre-screening questionnaire, English-speaking participants did not speak any other languages. By contrast, most of the Bangla-speaking participants reported speaking ⩾2 additional languages, such as Urdu. This is typical of South Asian populations and, as the learning and use of additional languages is often informal and contingent on social and functional contexts (Bhatia & Ritchie, 2006), we did not collect information about qualifications or levels achieved.

#### Final sample

Due to experimenter error, one additional English-speaking participant was recruited, resulting in a final *n* = 31. There were two Bangla-speaking participants who did not complete the full experiment, resulting in a final *n* = 28). In addition to the main sample, we also recruited an additional sample of Bangla speakers (*n* = 10; see details in section “Experimental procedure”). Participants were paid approximately £8 per hour for their time.

### Stimuli

Two bilingual English and Bangla speakers (1 female, 1 male) were recruited to produce the stimuli. We selected speakers who conducted daily life conversing in English, received secondary and university education in English, and had lived in the United Kingdom since early adolescence, but who primarily spoke Bangla during their childhood in Bangladesh. This strategy was chosen to prioritise so-called “native” pronunciation in Bangla, with the acknowledgement that operationalising primary and secondary languages (as is typically done in Western societies) is problematic (Holliday, 2006; Jaspal, 2010) and not necessarily applicable within South Asia or many other regions where plural multilingualism is the norm (Bhatia & Ritchie, 2006; Vulchanova et al., 2022). The speakers’ accents would likely be identified as South Asian English by the English-speaking sample, an umbrella term encompassing multiple dialects associated with immigrant and second or later generation communities throughout the United Kingdom (Jaspal, 2010).

Text prompts included the North Wind and the Sun story from Aesop’s Fables and other short compositions that were of a similar reading level, as well as spontaneous speech. The English text prompts are described in more detail in a previous work (MacIntyre & Scott, 2022), and translations to Bangla were devised by author R.A., who speaks Bangla as a primary language. At the time of preparation for this study, the university campus had not yet reopened following the COVID-19 pandemic, so the speakers recorded the speech stimuli at their residences using consumer grade microphones in a quiet space sampled at 48 kHz. The speech audio recordings were preprocessed using Audacity^®^ 3.0.0 (Audacity Team, 2023). Namely, loudness was normalised using root mean square and residual background noise was removed. Qualitatively, the sound of the audio recordings can be compared with a voice note or call made with a modern smartphone.

Following the form described by Deutsch et al. (2011), each stimulus consisted of a longer segment of continuous speech, followed by a shorter, repeating segment excerpted from the initial utterance. There were a total of 10 repetitions of the shorter segment, with repeats separated by a silent pause of 1,000 ms. From the full-length recordings, a total of 54 preliminary stimuli were initially generated. An example trial contains the complete utterance, “And, also, the fact that there are many opportunities, uh, for many people to come here to grow, to build a career,” with the repeating phrase, “for many people to come here.”

To evoke a range of ratings in the main study, pilot ratings were obtained from a bilingual sample (see Supplementary Materials for details) to generate a subset of stimuli ranging from highly speech-like to highly song-like. From the initial stimuli set, we took forward the eight highest-rated and the four lowest-rated Bangla and English stimuli (total n=24 stimuli, 12 per language). The mean duration of English repeated phrases was 1.62 s (SD=0.45) with 6.67 syllables (SD=2.18) syllables, and the mean duration of Bangla repeated phrases was 1.60 s (SD=0.64) with 7.08 (SD=2.69) syllables. Overall, the mean total trial duration, including both continuous and repeated segments, was 31.88 s (SD=6.46) . The stimuli can be accessed from https://osf.io/xvhjz/.

### Experimental procedure

The study protocol received approval from the relevant institutional ethics committee and all participants provided informed consent prior to enrolment in the experiment. The task order, for all participants, was speech-to-song illusion listening task, rhythm discrimination task, and demographics questionnaire. Participants were given the opportunity to take self-paced rests between experimental tasks. The median experimental session duration was approximately 40 min, with a system time limit of 60 min. The experimental materials can be viewed at https://app.gorilla.sc/openmaterials/777916. We did not carry out a formal headphone check, but provided clear instructions in the recruitment materials and task instructions that participants should use headphones and listen somewhere quiet.

#### Speech-to-song illusion listening task

Participants were first provided with a description of the illusion as well as experiment instructions. Trials consisted of listening to each stimulus in full and, after presentation ended, rating the stimulus on a 5-point Likert-type slider scale, with 1 representing *completely speech-like quality*, and 5 representing *completely song-like quality*. Each trial was initiated with a visual cue indicating that sound playback was about to start. Note that we did not collect baseline or pre-repetition ratings of the stimuli: This choice was made pragmatically to minimise experiment duration, as internet access and connection stability are highly variable within Bangladesh. Although well established in English-speaking populations, the specific role of repetition is unclear for Bangla speakers. To address this, we collected an additional sample of Bangla-speaking participants (n=10) who also contributed baseline ratings, thereby allowing us to confirm a change in response following stimulus repetition. The 24 stimuli were presented in four counterbalanced, pseudo-randomised trial orders, to which participants were randomly assigned.

#### Auditory rhythm discrimination task

To assess individual participants’ sensitivity to auditory sequential structure, we administered a two-alternative forced choice discrimination task that is described in MacIntyre and Scott (2022) and MacIntyre et al. (2023). Briefly, the rhythm discrimination task consisted of 40 trials, each containing two repetitions of an auditory rhythm followed by a short pause and the presentation of a second rhythm. Following stimulus presentation, participants were asked whether the latter rhythm was “Same” or “Different” from the first. The rhythm stimuli were each 3.20 s in duration and were composed of nine conga drum sounds separated by various configurations of the following inter-onset intervals: 5 ms × 200 ms; 2 ms × 400 ms; 1 ms × 600 ms; and 1 ms × 800 ms. Each recombination of intervals creates a unique rhythmic sequential pattern.

#### Demographics questionnaire

To quantify self-reported musical engagement, we designed a short questionnaire that could be phrased similarly across the English- and Bangla-speaking groups. It consisted of subjective estimates of music and dance activities, during childhood and at present, with responses chosen from seven possible answers. For example, participants were asked to select the answer that best described their current engagement with music:

1. Little to no music listening or training
2. Music listening but no training
3. Music listening and some informal participation (e.g., occasional classes)
4. Music listening and some training
5. Music listening and regular training
6. Music listening and regular training and ensemble (e.g., orchestra or jazz band)
7. Advanced amateur and/or professional-level training

The childhood music and childhood and adult dance responses were similarly worded and can be viewed at https://app.gorilla.sc/openmaterials/777916.

### Analysis

Descriptive statistics are aggregated over multiple responses within-participant, before summarising at the group level. We examined ratings in the illusion listening task using cumulative link mixed models (CLMMs). CLMMs are a class of regression models that can incorporate random effects (e.g., Participant; Stimulus) and were developed for ordered categorical data such as Likert-type scale responses (Christensen & Brockhoff, 2013; Taylor et al., 2023). These models work by treating the discrete outcome variable (i.e., Rating) as though its levels represent the thresholds or cut-off points of an underlying continuous distribution. Hence, larger predictor or coefficient estimates will be associated with a greater frequency of observations with higher ratings. We fit the CLMM using maximum likelihood estimation with Laplace approximation as implemented by the package ordinal (Christensen, 2011) in R 4.0.3 (R Core Team, 2021). We performed model selection using the Akaike information criterion (AIC; Burnham et al., 2011) and evaluated the statistical significance of fixed effects (predictor terms) with likelihood ratio tests. Random intercepts and slopes, which allow baseline and experimental outcomes to vary across participants or stimuli, were also fit. Pairwise comparisons were conducted using estimated marginal means with the emmeans package (Lenth, 2016). We use Pearson’s *r* for normally distributed continuous variables and Spearman’s Rho, a non-parametric measure of rank correlation, otherwise. Where applicable, *p*-values are adjusted using the False Discovery Rate correction. Confidence intervals for descriptive and bivariate analyses were estimated by using bootstrapped sampling with replacement (*n* = 1,000 iterations).

## Results

### Baseline ratings

To verify that stimulus repetition led to higher or more song-like scores, we examined responses from a separate sample of Bangla speakers (n=10) who produced both baseline and post-repetition ratings. A paired *t*-test confirmed that post-repetition ratings ( M=2.56 , SD=1.10 , Min = 1.50 Min=1.50 , Max=4.20 ) were significantly higher than pre-repetition ratings ( M=2.16 , SD=1.07 , Min=1.30 , Max=3.90 ; t(23)=6.07 , p<.001 ). The post-repetition change in average ratings ranged from Min=0.30 to Max=1.28 . With respect to Stimulus Language, mean post-repetition change in ratings was 0.47 (SD=0.27) for English stimuli and 0.35 (SD=0.33) for Bangla stimuli. An independent *t*-test showed this difference was non-significant (p=.340) . Finally, we correlated average post-repetition ratings, by stimulus, between this sample and the full sample of Bangla speakers. We found r=.62 (95% CI=[0.34,0.79] , p=.001 ), indicating moderate-to-strong reliability across samples.

### Full sample

Summary statistics and Spearman’s rank correlations regarding Rhythm Task Score and self-reported measures are shown in Table 1. For the most part, differences between English- and Bangla-speaking participants were small. In particular, English and Bangla speakers performed similarly in the Rhythm Discrimination Task (p=.410) . Although statistically non-significant, there was a tendency for Bangla speakers to report slightly less Childhood Musical Experience (p=.056) and slightly more Current Dance Experience (p=.090) in comparison to English speakers. The numerically strongest correlation was between Rhythm Task Score and reported Childhood Musical Experience ( $r_{s}=.34$ , p=.009 ). Participants’ childhood and current experiences of music and dance were also weakly correlated, respectively. Approximately 80 % of participants rated their Current Musical Experience as ≤2 (“Music listening but no training”); hence, the absence of an association between this measure and Rhythm Task Score ( $r_{s}=.05$ , p=.710 ) probably reflects a lack of coverage for currently musically active participants, more so than a true dissociation between these traits.

**Table 1.** Summary statistics and correlations pertaining to self-reported musical experience and performance in the rhythm discrimination task.

	English speakers (n=31)			Bangla speakers (n=28)			Mann–Whitney *U* test		
	Median	MAD	IQR	Median	MAD	IQR	*U*	*p*	*r*
Rhythm Task Score	72.5	10	23.13	68.75	10	37.5	0.83	.407	.11
Current musical experience	2	0	0.75	2	0	0	1.33	.185	.17
Childhood musical experience	2	1	2	2	0.5	1	1.91	.056	.25
Current dance experience	1	0	1	2	0	1	−1.72	.085	−.22
Childhood dance Experience	2	1	1	1	1	1	0.89	.372	.12
	Spearman’s rank correlation (*p*)								
	Rhythm task		Childhood music		Current music		Childhood dance		Current dance
Rhythm Task	1		0.34 (0.009)		0.05 (0.710)		−0.18 (0.171)		−0.28 (0.029)
Childhood music			1		0.3 (0.022)		0.13 (0.311)		0.00 (0.981)
Current music					1		0.13 (0.342)		0.2 (0.134)
Childhood dance							1		0.32 (0.015)
Current dance									1

MAD: median absolute deviation; IQR: inter-quartile range.

Rhythm Task Score is calculated as % Correct. Self-reported measures are on a scale of 1−7 . *p*-values are uncorrected.

### CLMM of ratings

**Figure 1.**
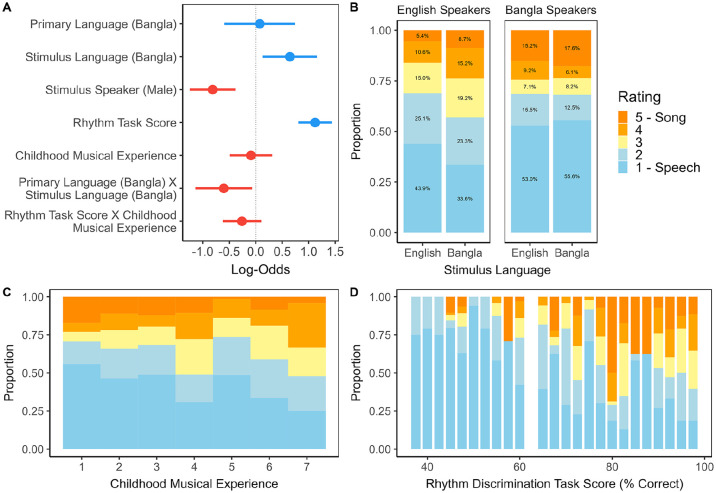
Panel A: Dot-and-whisker plot depicting standardised regression coefficient estimates with 95% confidence intervals from the cumulative link mixed effects model of ratings (scale of 1 − 5, where 1 = *speech-like* and 5= *song-like*) in the Speech-to-Song-Illusion listening task. The model reference levels were English Primary Language, English Stimulus Language, and Female Speaker Stimulus Speaker. The blue and red marker colours indicate positive and negative effect directions, respectively. Panel B: Stacked bar plot indicating the proportion of ratings by Primary Language and Stimulus Language. Panel C: Stacked bar plot indicating the proportion of ratings by self-reported Childhood Musical Experience. Panel D: Stacked bar plot indicating the proportion of ratings by score (percent correct) in the Auditory Rhythm Discrimination task.

We analysed the ratings of illusion stimuli using CLMMs, finalising the predictors and random effects based on AIC. Full details of this model term selection and the final model are provided in Supplemental Appendix, Tables 3–4. The standardised coefficients included in the final model are plotted in Figure 1, Panel A.

The main effect of Stimulus Speaker (Log – Odds = -0.81, 95% CI = [−1.24,−0.38] , z=−3.71 , p=.001 ) indicated that the Female speaker ( M=2.38 , SD=0.89 ) tended to evoke more song-like ratings than the Male speaker ( M=1.89 , SD=0.67 ). Although this predictor was included as a control or covariate in the full model, we examine this discrepancy further in the exploratory acoustic analysis. With regard to the effects of interest, Primary Language was non-significant (Log - Odds =0.08 , 95% CI = , z=0.22 , p=.822 ): On average, Bangla speakers ( M=2.25 , SD=0.70 ) and English speakers ( M=2.17 , SD=0.85 ) gave similar ratings. In addition, the main effect of Stimulus Language (Log-Odds =0.64 , 95% [0.13,1.16] , z=2.47 , p=.014 ), though significant, did not improve model fit ( $\chi^{2}=2.46$ , *p* =.117 ). There was, however, a statistically significant interaction between Stimulus Language and Primary Language ( Log−Odds=−0.60 , 95% CI = [−1.14,−0.07] , z=−2.20 , p=.028 ). Pairwise tests (Supplemental Appendix, Table 4) showed that, whereas Bangla-speaking participants did not differentiate between Bangla and English stimuli ( Log−Odds=0.02 , 95% CI=[−0.28,0.32] , $p_{\mathrm{fdr}}=.877$ ), English speakers tended to rate Bangla stimuli as more song-like ( M=2.41 , SD=0.79 ) than English stimuli ( M=2.08 , SD=0.71 ; Log−Odds=0.32 , 95% CI=[0.03,0.61] , $p_{\mathrm{fdr}}=.014$ , d=0.31 , 95% CI = [0.06,0.56] ; Figure 1, Panel B). With regard to the numeric predictors, higher Rhythm Task Score was associated with increasingly song-like ratings ( Log−Odds=0.09 , 95% CI = [0.05,0.12] , z=4.71 , p<.001 Figure 1, Panel C). Participants with the lowest scores ( <60.83 , n=20 ) gave an average rating of *M* =1.54 (SD=0.55) . Those with medium scores ( 60.83−77.50 , *n* =20 ) gave an average rating of *M* =2.30 ( SD=0.56 ). Finally, participants with the highest scores ( >77.50 , *n* = 19) gave an average rating of M=2.82 (SD=0.60) . Individually, Rhythm Task Score predicted higher mean ratings both in English speakers ( $r_{s}=.63$ , 95% CI = [0.34,0.82] , $p_{fdr}<.001$ ) and Bangla speakers ( $r_{s}=.75$ , 95% CI = [0.56,0.87] , $p_{fdr}<.001$ ).

The only self-reported measure to improve model fit was Childhood Musical Experience, which was correlated with Rhythm Task Score $\left(r_{s}=.34\right)$ . Including an interaction term between the continuous predictors increased model likelihood ( $\chi^{2}=26.30$ , p<.001 ), but the parameter was non-significant ( Log−Odds=−0.01 , 95% CI = [−0.02,0.00] , z=−1.40 , p=.163 ).

#### Agreement of ratings

We sought to quantify how consistently participants rated the stimuli, in terms of individuals as well as Primary Language groups. Agreement was estimated using Kendall’s coefficient of concordance (*W*), a non-parametric statistic that is bounded between 0 (no agreement) and 1 (perfect agreement). Within English speakers, *W* =0.25 ( $\chi^{2}=150.16$ , p<.001 ), indicating weak agreement between individual participants. For Bangla speakers, W=0.15 , corresponding to relatively poor agreement between raters ( $\chi^{2}=62.10$ , p<.001 ). Finally, we aggregated ratings at the level of Primary Language to determine concordance between English and Bangla speakers. In this case, W=0.75 ( $\chi^{2}=34.50$ , p<.001 ), suggesting moderately good agreement between the Primary Language groups. Hence, although individual participants vary in their response to specific stimuli, linguistic or cultural differences do not appear to play a substantive role in this process at the group level.

### Exploratory acoustic analysis of stimuli

Previous studies investigating the acoustic correlates of the speech-to-song illusion show that pitch (F0) information can predict the extent to which speech is perceived as song-like (Falk et al., 2014; Groenveld et al., 2020; Rathcke et al., 2021). Regularity of syllabic timing may also affect the illusion (Falk et al., 2014; Tierney et al., 2018). Although the current study was not specifically designed to test the effects of acoustic features on the illusion, we were interested in whether any stimulus-specific properties would predict ratings. Moreover, the CLMM revealed a main effect of Stimulus Speaker, with the female speaker receiving higher ratings in general. We, therefore, also sought to identify possible acoustic differences that could shed light on the speaker effect.

#### Acoustic features

One of the key acoustic differences proposed to separate speech from song is the presence and consistency of sustained pitch (De Medeiros et al., 2021; Natke et al., 2003; Zatorre & Baum, 2012). To quantify pitch variability in our stimuli, we estimated and manually corrected the fundamental frequency in Praat (Boersma & Weenink, 2009). We then calculated two summary variables: First, we found the *SD* of pitch (Hz) within each voiced portion of the audio, and then took the average of *SD* values over the complete phrase. This statistic, which we term Pitch Variability, captures moment-to-moment stability. Second, we took the mean pitch from each voiced 40 ms-segment of the utterance, and calculated the absolute difference (Hz) over successive portions. This measure, Pitch Change Over Time, expresses pitch change over the course of the full phrase.

Next, we estimated the stimulus harmonic ratio using the Audio Toolbox in MATLAB (The MathWorks Inc., 2020). The harmonic ratio expresses the amount of harmonic energy relative to total energy in a sound. It is higher for voiced, in comparison to unvoiced, speech sounds, and is related to the perceptual qualities of vocal roughness (Eskenazi et al., 1990; Yumoto et al., 1982) and sonority (Komatsu et al., 2002). In particular, sonority was previously implicated in a study investigating the speech-to-song illusion (Rathcke et al., 2021). Hence, at the level of utterance, we calculated Mean Harmonic Ratio, as well as the proportion of 40 ms-windows where the harmonic ratio was high, arbitrarily set as >.80 (Proportion High Harmonic Ratio).

Finally, we used two measures of syllabic timing to quantify the differentiation of syllabic stress in our stimuli: the nPVI (Nolan & Asu, 2009), which is higher for utterances with greater durational contrast between successive syllables; and the coefficient of variation, wherein the standard deviation of inter-syllabic timing is normalised by the mean inter-syllabic interval. We calculated the nPVI and CV based on manually annotated vowel onsets, which were defined as the point at which upper formants became visible in the voiced parts of the spectrogram. Summary statistics by Speaker and Language are given in Table 2.

**Table 2.** Summary statistics of acoustic features by stimulus speaker and language.

	*M* (*SD*)			
	English		Bangla	
	Female speaker	Male speaker	Female speaker	Male speaker
Ratings (full sample)	2.34 (0.39)	1.75 (0.27)	2.41 (0.40)	1.99 (0.46)
Pitch (Hz)	218.72 (17.6)	117.71 (6.03)	221.91 (15.8)	120.4 (18.74)
Pitch variability (Hz)	9.97 (4.39)	8.55 (1.04)	4.30 (2.30)	4.30 (2.30)
Pitch change over time (Hz)	79.55 (54.33)	49.39 (34.27)	96.88 (32.03)	42.79 (34.08)
Harmonic ratio	0.75 (0.05)	0.64 (0.06)	0.72 (0.04)	0.59 (0.09)
Proportion harmonic ratio >0.80	0.58 (0.11)	0.13 (0.13)	0.48 (0.11)	0.14 (7.07)
nPVI vowel onsets	55.75 (18.25)	53.61 (0.17)	50.96 (14.47)	55.96 (19.00)
CV vowel onsets	0.42 (00.17)	0.33 (0.11)	0.40 (0.21)	0.43 (0.20)

CV: coefficient of variation; nPVI: normalised pairwise variability index.

#### Linear model of mean ratings

We modelled mean stimulus rating by primary language group using ordinary least squares linear regression. Assuming the categorical term Primary Language, additional model terms were selected with respect to AIC and the add1 and drop1 functions in R, which test the significance of the addition or removal of individual predictors using *F*-tests. Besides the acoustic features, we also attempted to fit Stimulus Speaker and Stimulus Language, as well as their interactions with the continuous acoustic variables.

**Figure 2.**
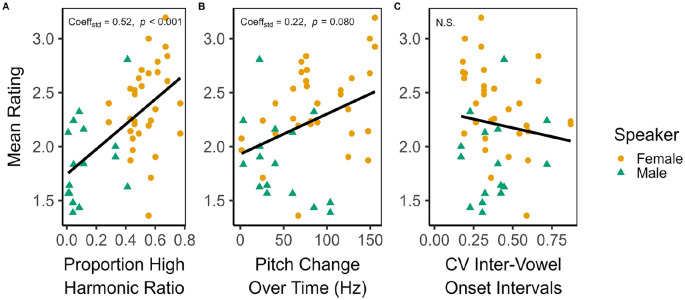
Standardised coefficients in the linear model predicting mean stimulus rating (scale of, 1−5 where 1= *speech-like* and 5= *song-like*) in the full sample of English and Bangla speakers: Panel A, Proportion speech harmonic ratio, describing the duration of speech with high harmonic-to-noise ratio relative to the total duration of the stimulus; Panel B, Pitch change over time (Hz), the sum of absolute change in mean pitch across the voiced parts of each stimulus; and Panel C, the coefficient of variation of inter-vowel onset intervals, an estimate of the variability of syllabic timing.

The final model included, in addition to Primary Language, two covariates: Proportion High Harmonic Ratio and Pitch change over time, $R^{2}=.40$ , F(3,44)=9.65 , p<.001 , AIC = 45.44. As the female speaker was generally associated with higher harmonic ratio values than the male speaker, we also tested several models that also incorporated Stimulus Speaker as a predictor. A model containing Stimulus Speaker in place of Proportion high harmonic ratio had AIC AIC=47.43 . The addition of Stimulus Speaker to a model containing Proportion high harmonic ratio also resulted in a worse fit ( p=.946 , =51.73 ). Conversely, adding Proportion high harmonic ratio to a model containing Stimulus Speaker significantly improved fit ( p=.018 ). As such, Proportion high harmonic ratio is a more powerful explanatory variable than Stimulus Speaker. Increases in the proportion of high harmonic ratio segments was associated with more song-like mean ratings ( Estimate=1.03 , 95% CI=[0.54,1.52] , t(44)=4.24 , p<.001 , $F_{partial}^{2}=.57$ ). There was also a non-significant, positive trend of Pitch change over time leading to more song-like ratings (Estimate =0.002 , 95% CI  = [−0.000,0.004] , t(44)=1.79 , p=.080 , $F_{partial}^{2}=.07$ ). The effect of Primary Language was non-significant (Estimate −0.10 , 95% CI = [−0.31,0.12] , t(44)=−0.92 , p=.363 , $F_{partial}^{2}=0.02$ ). We plot Proportion high harmonic ratio and Pitch change over time with standardised model coefficients in Figure 2, Panels A and B. For comparison, CV of inter-vowel onset intervals is also shown in Panel C. This and the other acoustic features, including nPVI and Pitch variability, did not appear to have a strong relationship with mean ratings.

#### Additional Bangla-speaking sample

To confirm the findings of the exploratory acoustic analysis, we ran another linear regression using the post-repetition ratings provided by the separate sample of Bangla speakers (n=10) . The model specification was the same as before, but excluding the Primary Language term. This analysis confirmed that mean ratings were increasingly song-like with greater Proportion high harmonic ratio Estimate=1.89 , 95% CI = [0.63,3.16] , t(21)=3.11 , p=.005 , $F_{partial}^{2}=0.62$ ). However, the positive, non-significant trend of Pitch change over time that we observed in the first model was absent ( Estimate=0.00 , 95% CI = [0.00,0.01] , t(21)=1.15 , p=.263 , $F_{partial}^{2}=0.06$ ).

Given that this sample also provided baseline or pre-repetition responses, we could additionally examine the relationship between the acoustic features and pre-illusion ratings. Harmonic ratio was associated with more song-like ratings of baseline stimuli, but the effect was smaller than for post-repetition responses ( Estimate=1.82 , 95% CI = [0.48,3.15] , t(21)=2.84 , p=.010 , $F_{partial}^{2}=0.43$ ). Pitch change over time was non-significant (*p* = 0.863). Finally, we modelled the mean difference in ratings (i.e., post-repetition—pre-repetition), which we calculated first within participant before aggregating by stimulus at the group level. Interestingly, although there was no relationship with Harmonic ratio ( Estimate=0.08 , 95% CI = [−0.46,0.63] , t(21)=0.32 , p=.752 , $F_{partial}^{2}=0.05$ ), we observed a positive trend of Pitch change over time ( Estimate=0.003 , 95% CI = [0.00,0.01] , t(21)=2.18 , p=.041 , $F_{partial}^{2}=0.23$ ). Hence, Harmonic ratio appears to affect the song-like quality of the stimuli before and after repetition, but Pitch change over time may interact with the perceptual transformation. In light of the small sample size and incidental nature of this result, however, this potential link should be confirmed in a planned larger study.

## Discussion

The current study was motivated to examine the speech-to-song illusion in speakers of Bangla, an Indo-Aryan language that is spoken by hundreds of millions of people worldwide, but remains underrepresented in speech perception research. We predicted that Bangla speakers would report experiencing the illusion to a similar extent as English speakers. This was born out of the data, but our secondary hypothesis for a cross-linguistic interaction was only partly confirmed. Namely, whereas English-speaking listeners gave Bangla stimuli more song-like ratings than English stimuli, the Bangla-speaking listeners did not distinguish between English and Bangla stimuli in their ratings—an outcome that is complicated to interpret, due to ambiguities concerning the Bangla speakers’ familiarity with English. In the case of possible baseline differences in music engagement, we also collected demographic reports and administered a non-verbal auditory rhythm discrimination task. Although we observed no differences—neither in subjective reports nor in objective task performance—between English and Bangla speakers, individual perceptual sensitivity to temporal sequences was predictive of increasingly song-like ratings across both groups. Finally, we undertook an exploratory acoustic analysis of our stimuli to identify physical correlates of the illusion. In particular, the proportion of high harmonic ratio segments across an utterance predicted its mean rating for both language groups, a result that we confirmed in an independent sample of Bangla speakers, but which should also be followed up in a hypothesis-driven study. We discuss these findings in more detail to follow.

### Cross-linguistic effects

The perceptual and cognitive mechanisms underlying the speech-to-song illusion are a topic of active discussion. One theory focuses on “semantic satiation,,” positing that the repetition of the speech stimulus leads to its meaning or informational content to become redundant, at which point acoustic, rather than linguistic, cues dominate in perceptual salience (see Tierney et al., 2018, for further discussion). An alternative approach draws on Node Structure Theory from linguistics, whereby word- and sentence-level “nodes” become satiated, allowing the listener to attend to lower-level features, such as syllables (Vitevitch et al., 2021, 2023). Finally, another framework considers phonological access (rather than comprehension) of listeners, hypothesising that unfamiliar languages with sound inventories more distant from a listener’s own language will be more likely to evoke the illusion (Margulis et al., 2015). In line with previous work (Castro et al., 2018; Jaisin et al., 2016; Margulis et al., 2015), English speakers in the current study rated Bangla stimuli as more song-like, but this effect was modest, and we found no reciprocal difference in the ratings of Bangla-speaking listeners.

There are two important factors that challenge interpretation here. First, although we made an effort to recruit Bangla speakers with minimal knowledge of English, the populations we reached via online testing are likely to encounter English on a daily basis, as discussed in the Introduction and “Materials and methods” sections. Besides exposure from primary education, media, and advertising, a substantial number of English loanwords proliferate throughout Bangla (e.g., daktar, doctor; tebil, table). It is, therefore, possible that the English stimuli were at least partially comprehensible to the Bangla-speaking participants. In which case, the current results align with other studies where the illusion language is non-primary, but familiar to listeners (Rathcke et al., 2021; Tierney et al., 2017). The second source of ambiguity is that the speakers who provided the stimuli in the current study, though fluently bilingual, spoke Bangla-accented English. If phonological access does modulate the speech-to-song illusion (Castro et al., 2018; Margulis et al., 2015), we imagine that differences in phonemic and prosodic inventory between the two languages would be minimised when the stimuli are spoken with an accent familiar to Bangla-speaking listeners, potentially resulting in similar ratings across stimulus languages.

Another possibility is that listening strategies differed across the two language groups. For example, Bangla is noted for its “regular repeating patterns [in the] pitch contour,” which can be contrasted with the “lack of such regularity in English” (Yu et al., 2014, p. 1131). In other words, intonation is a particularly salient feature for Bangla-speaking listeners (Khan, 2014). Potentially, enhanced sensitivity to pitch contour information could enhance experience of the illusion irrespective of language understanding. This possibility could be tested in future work by applying manipulations to the pitch contour specifically (Groenveld et al., 2020; Tierney et al., 2018). It would also cohere with recent evidence that even short-term experience with the illusion can alter participants’ readiness for perceiving the transformation in novel stimuli (Kubit et al., 2024; Soehlke et al., 2022). Nevertheless, English and Bangla speakers showed good agreement in their ratings across stimuli, and the exploratory acoustic analysis suggests that physical properties of the speech signal affect their experience of the illusion in similar ways, consistent with recent evidence from a cross-linguistic sample of English, Mandarin, and Cantonese speakers (Kachlicka et al., 2024). Thus, we find more commonalities than differences when comparing the two groups.

### Sensitivity to auditory rhythmic sequences

The music demographics questionnaire and the rhythm discrimination task were included in the current experiment to account for possible baseline differences between language groups. As it turned out, our samples of English and Bangla speakers were well-matched on these measures, but the data afforded us the opportunity to examine individual sensitivity to auditory sequential structure. As a predictor, the rhythm discrimination task was strongly indicative of listeners’ song-like ratings, beyond reported childhood musical experience. This association aligns with previous work investigating individual differences in the illusion (Tierney et al., 2021). It may also speak of an emerging, broader literature linking non-verbal rhythm skills to speech and language abilities (Borrie et al., 2017; Fiveash et al., 2021; MacIntyre & Scott, 2022; Tierney et al., 2017). A rhythm-specific interpretation, however, is limited by the fact that we did not administer any other auditory tasks. As described in the “Materials and methods” section, we focused on rhythm sequence discrimination as this involves the online processing of both temporal and ordinal information (i.e., the timing of onsets and patterning of intervals). We did not test participants’ ability in the spectral domain, for example, by administering a melody discrimination task. Hence, we cannot be sure that rhythm discrimination, and not auditory processing more generally, is related to the illusion. At the least, our results underscore the utility of collecting objective measures, rather than relying on reported years of formal training, to estimate musical or non-verbal auditory aptitude (Madsen et al., 2019; Mankel & Bidelman, 2018). Participants reporting little to no present or past musical experience scored highly in the rhythm discrimination task. This places the current data in line with a recent study showing that self-identifying non-musicians are capable of achieving scores comparable to—or even better than—musicians in some rhythm tasks (Correia et al., 2023).

### Acoustic correlates of song-like stimuli

Previous speech-to-song illusion experiments explicitly assessed whether certain acoustic properties can lead to more intense experience of the illusion. For instance, Rathcke et al. (2021, p. 507) proposed that the “increased amount of transmittable pitch information fosters the transformation” from speech to song. In that study, the authors operationalised the perceptual availability of pitch using sentence sonority, a phonological feature that characterises the prominence of speech sounds, with open vowels being most sonorous, and voiceless stops and fricatives being least sonorous. They found that high-sonority sentences received more song-like ratings (Rathcke et al., 2021). Sonority is a linguistic, as opposed to acoustic, concept and we did not undertake manual annotation of sonority in our stimuli; however, the harmonic ratio is directly related to the vibration of the vocal cords, so we would expect that this acoustic feature tracks closely with sonority. Multiple linear regression revealed a moderately strong correlation between the proportion of high harmonic ratio speech and more song-like ratings across independent samples. Although the two speakers incidentally differed on this feature, model comparisons confirmed that the presence of harmonic ratio better predicted ratings than speaker identity. Notably, in the additional sample of Bangla speakers, we found that harmonic ratio also increased the song-like impression of pre-illusion ratings, and this acoustic feature did not predict pre- and post-repetition differences in responses. In other words, although harmonic ratio influences song-like ratings, it does not contribute to a perceptual transformation per se.

In the full sample only, we observed a statistically non-significant association between song-like responses and the variance of pitch across an utterance. This same acoustic feature significantly correlated with differences between pre- and post-repetition ratings in the separate sample of Bangla speakers, but we interpret this incidental finding with caution. Future work with a larger number of speakers producing acoustically controlled stimuli should clarify whether harmonic ratio and other, pitch-related features are predictive of ratings in the illusion. We found no trend linking the regularity of syllabic timing to song-like ratings. This accords with other studies finding that speech rhythm at this level does not strongly shape the illusion (Falk et al., 2014; Tierney et al., 2018). However, we used two simplistic measures to estimate temporal regularity: namely the CV and nPVI of inter-vowel onset intervals. Isochronous or equally timed syllable rhythm is atypical under most speaking conditions (Turk & Shattuck-Hufnagel, 2013), but that does not mean that speech rhythm is, in general, unpredictable or arrhythmic. It is possible that other sources of temporal structure in the illusion stimuli may have influenced the perceptual transformation, such as the presence of metrical patterns or musical beat-like cues (Falk et al., 2014; Kachlicka et al., 2024). Similarly, our measures of pitch variability may not map well onto the percept of sustained tonality (De Medeiros et al., 2021). Future work investigating cross-linguistic differences in prosody perception should use more robust, complementary techniques to examine these potentially more subtle effects.

## Conclusion

Perception is an active and cognitive process, rather than the passive, unidirectional flow of information. The speech-to-song and other illusions may offer important insights into how top–down or endogenously originating factors shape subjective sensory experience, but this larger goal demands greater sample diversity, as well as taking linguistic, cultural, and other contextual factors into account. The current study is a preliminary step to describing the speech-to-song illusion phenomenon in a Bangla-speaking, South Asian population; however, the sample still only represents young, digitally connected populations (Ghai, 2021). Hence, although we observed cross-linguistic effects in English-speaking listeners only, it is possible that this interaction reflects the asymmetrical nature of global English exposure, more so than particular linguistic differences between English and Bangla. This unresolved question emphasises the need for further studies comparing a wider variety of listeners recruited from typologically distinct languages. We can conclude that the present data speak of the relevance of individual differences in auditory perception as potentially key to the illusion in both English- and Bangla-speaking participants. Future work should clarify our incidental findings of commonalities, as well as identify the basis of possible differences, across these and other diverse languages.

## Supplemental Material

sj-docx-1-qjp-10.1177_17470218241293627 – Supplemental material for Cross-linguistic effects of the speech-to-song illusion in speakers of Bangla and English Supplemental material, sj-docx-1-qjp-10.1177_17470218241293627 for Cross-linguistic effects of the speech-to-song illusion in speakers of Bangla and English by Rakhi Akter and Alexis Deighton MacIntyre in Quarterly Journal of Experimental Psychology
