# Supplementary material for: Cross-linguistic effects of the speech-to-song illusion in speakers of Bangla and English
Source: Q J Exp Psychol (Hove). 2024 Dec 9;78(9):1852–65. doi: 10.1177/17470218241293627 (PMC12335629; doi:10.1177/17470218241293627)
Supplement: sj-docx-1-qjp-10.1177_17470218241293627 – Supplemental material for Cross-linguistic effects of the speech-to-song illusion in speakers of Bangla and English [file sj-docx-1-qjp-10.1177_17470218241293627.docx]

## Appendix

### Model Selection

Table 3

*Model selection for the dependent variable of Response (Rating $1-5$) in the speech-to-song illusion listening task. * Denotes the final model.*

| Model | Formula | AIC |
| --- | --- | --- |
| 1 | (1 | Participant) | 3644.02 |
| 2 | (1 | Participant) + (1 | Stimulus) | 3568.09 |
| 3 | Primary Language + (1 | Participant) + (1 | Stimulus) | 3569.39 |
| 4 | Primary Language + Stimulus Language + (1 | Participant) + (1 | Stimulus) | 3569.08 |
| 5 | Primary Language × Stimulus Language + (1 | Participant) + (1 | Stimulus) | 3562.84 |
| 6 | Primary Language × Stimulus Language + (Stimulus Language | Participant) + (1 | Stimulus) | 3562.12 |
| 7 | Primary Language × Stimulus Language + Stimulus Speaker + (Stimulus Language | Participant) + (1 | Stimulus) | 3552.70 |
| 8 | Primary Language × Stimulus Language × Stimulus Speaker + (Stimulus Language | Participant) + (1 | Stimulus) | 3552.60 |
| 9 | Primary Language × Stimulus Language + Stimulus Speaker + (Stimulus Language × Stimulus Speaker | Participant) + (1 | Stimulus) | 3559.73 |
| 10 | Primary Language × Stimulus Language + Stimulus Speaker + (Stimulus Language | Participant) + (Primary Language | Stimulus) | 3553.95 |
| 11 | Primary Language × Stimulus Language + Stimulus Speaker + Rhythm Task Score + (Stimulus Language | Participant) + (1 | Stimulus) | 3542.97 |
| 12 | Primary Language × Stimulus Language + Stimulus Speaker + Rhythm Task Score + Current Musical Experience + (Stimulus Language | Participant) + (1 | Stimulus) | 3544.21 |
| 13 | Primary Language × Stimulus Language + Stimulus Speaker + Rhythm Task Score + Childhood Musical Experience + (Stimulus Language | Participant) + (1 | Stimulus) | 3541.72 |
| 14 | Primary Language × Stimulus Language + Stimulus Speaker + Rhythm Task Score + Current Dance Experience + Current Dance Experience + (Stimulus Language | Participant) + (1 | Stimulus) | 3544.11 |
| 15 | Primary Language × Stimulus Language + Stimulus Speaker + Rhythm Task Score + Childhood Dance Experience + (Stimulus Language | Participant) + (1 | Stimulus) | 3542.68 |
| 16 | Primary Language × Stimulus Language + Stimulus Speaker + Rhythm Task Score × Primary Language + Childhood Musical Experience + (Stimulus Language | Participant) + (1 | Stimulus) | 3548.23 |
| 17 | Primary Language × Stimulus Language + Stimulus Speaker + Rhythm Task Score + Childhood Musical Experience × Primary Language + (Stimulus Language | Participant) + (1 | Stimulus) | 3543.74 |
| 18* | Primary Language × Stimulus Language + Stimulus Speaker + Rhythm Task Score × Childhood Musical Experience + (Stimulus Language | Participant) + (1 | Stimulus) | 3517.42 |

### Cumulative Link Mixed Model of Ratings

Table 4

*Model formula: Rating ~ Primary Language × Stimulus Language + Stimulus Speaker + Rhythm Task Score × Childhood Musical Experience + (Stimulus Language | Participant) + (1 | Stimulus). Confidence intervals for estimates are computed from profile likelihood functions*.

|  |  |  |  | Wald Test | | Likelihood | |
| --- | --- | --- | --- | --- | --- | --- | --- |
| Ratio Test | |
| **Parameters** | Log-Odds | SE | 95% CI | *z* | *p* | $\chi^{2}$ | *p* |
| Threshold (Intercept): 1|2 | 5.65 | 1.43 | [2.85, 8.45] | 3.96 | $<0.001$ |  |  |
| Threshold (Intercept): 2|3 | 6.83 | 1.43 | [4.02, 9.64] | 4.77 | $<0.001$ |  |  |
| Threshold (Intercept): 3|4 | 7.71 | 1.44 | [4.89, 10.52] | 5.37 | $<0.001$ |  |  |
| Threshold (Intercept): 4|5 | 8.67 | 1.44 | [5.85, 11.49] | 6.02 | $<0.001$ |  |  |
| Primary Language | 0.08 | 0.34 | [-0.59, 0.74] | 0.22 | 0.822 | 0.95 | 0.331 |
| Stimulus Language | 0.64 | 0.26 | [0.13, 1.16] | 2.47 | 0.014 | 2.46 | 0.117 |
| Stimulus Speaker | -0.81 | 0.22 | [-1.24, -0.38] | -3.71 | $<0.001$ | 11.68 | 0.001 |
| Rhythm Task Score | 0.09 | 0.02 | [0.05, 0.12] | 4.71 | $<0.001$ | 13.81 | $<0.001$ |
| Childhood Musical Experience | 0.58 | 0.55 | [-0.49, 1.66] | 1.06 | 0.289 | 3.25 | 0.071 |
| Primary Language × Stimulus Language | -0.60 | 0.27 | [-1.14, -0.07] | -2.20 | 0.028 | 4.62 | 0.032 |
| Rhythm Task Score × Childhood Musical Experience | -0.01 | 0.01 | [-0.02, 0.00] | -1.40 | 0.163 | 26.30 | $<0.001$ |

| **Random Effects** |  |  |  |  |  |  |  |
| --- | --- | --- | --- | --- | --- | --- | --- |
| Within-Group Variance (SD) |  | 3.29 (1.81) |  |  |  |  |  |
| Between-Group Variance (SD) |  |  |  |  |  |  |  |
|  | Intercept (Participant) | 1.18 (1.09) |  |  |  |  |  |
|  | Intercept (Stimulus) | 0.23 (0.48) |  |  |  |  |  |
|  | Slope (Primary Language × Stimulus Language) | 0.35 (0.59) |  |  |  |  |  |
| Correlations |  |  |  |  |  |  |  |
|  | Participant × Stimulus Language | -0.49 |  |  |  |  |  |
| Groups |  |  |  |  |  |  |  |
|  | Participant | 59 |  |  |  |  |  |
|  | Stimulus | 24 |  |  |  |  |  |
| Observations |  | 1401 |  |  |  |  |  |

| **Model Metrics** | | | | | | | |
| --- | --- | --- | --- | --- | --- | --- | --- |
| AIC | BIC | R^2^ (Conditional) | R^2^ (Marginal) | ICC | RMSE |  |  |
| .42 | 3596.09 | 0.45 | 0.24 | 0.24 | 2.41 |  |  |

Contrast of Stimulus Language within Primary Language

Table 5

*Contrast of estimated marginal means (Rating) in the Speech-to-Song-Illusion listening task by Stimulus Language within Primary Language. P-values are False Discovery Rate-corrected.*

| Contrast: Stimulus Language | | At: Primary Language | Estimate | 95% CI | SE | *z* | *p* |
| --- | --- | --- | --- | --- | --- | --- | --- |
| English | Bangla | English | 0.32 | $[0.03,0.61]$ | 0.13 | $2.47$ | 0.014 |
| English | Bangla | Bangla | 0.02 | $[-0.28,0.32]$ | 0.14 | 0.16 | 0.877 |
